# Functional Characterization of Rhoptry Kinome in the Virulent *Toxoplasma gondii* RH Strain

**DOI:** 10.3389/fmicb.2017.00084

**Published:** 2017-01-24

**Authors:** Jin-Lei Wang, Ting-Ting Li, Hany M. Elsheikha, Kai Chen, Wei-Ning Zhu, Dong-Mei Yue, Xing-Quan Zhu, Si-Yang Huang

**Affiliations:** ^1^State Key Laboratory of Veterinary Etiological Biology, Key Laboratory of Veterinary Parasitology of Gansu Province, Lanzhou Veterinary Research Institute, Chinese Academy of Agricultural SciencesLanzhou, China; ^2^Faculty of Medicine and Health Sciences, School of Veterinary Medicine and Science, University of Nottingham, Sutton Bonington CampusLoughborough, UK; ^3^College of Animal Science and Veterinary Medicine, Shandong Agricultural UniversityTai’an, China; ^4^College of Animal Science and Veterinary Medicine, Heilongjiang Bayi Agricultural UniversityDaqing, China

**Keywords:** *Toxoplasma gondii*, CRISPR-Cas9 system, rhoptry proteins (ROPs), virulence factors, gene knockout, host–pathogen interaction

## Abstract

*Toxoplasma gondii* is an obligatory intracellular apicomplexan protozoan which can infect any warm-blooded animal and causes severe diseases in immunocompromised individuals or infants infected in utero. The survival and success of this parasite require that it colonizes the host cell, avoids host immune defenses, replicates within an appropriate niche, and exits the infected host cell to spread to neighboring non-infected cells. All of these processes depend on the parasite ability to synthesis and export secreted proteins. Amongst the secreted proteins, rhoptry organelle proteins (ROPs) are essential for the parasite invasion and host cell manipulation. Even though the functions of most ROPs have been elucidated in the less virulent *T. gondii* (type II), the roles of ROPs in the highly virulent type I strain remain largely un-characterized. Herein, we investigated the contributions of 15 ROPs (ROP10, ROP11, ROP15, ROP20, ROP23, ROP31, ROP32, ROP33, ROP34, ROP35, ROP36, ROP40, ROP41, ROP46, and ROP47) to the infectivity of the high virulent type I *T. gondii* (RH strain). Using CRISPR-Cas9, these 15 ROPs genes were successfully disrupted and the effects of gene knockout on the parasite’s ability to infect cells *in vitro* and BALB/c mice *in vivo* were investigated. These results showed that deletions of these ROPs did not interfere with the parasite ability to grow in cultured human foreskin fibroblast cells and did not significantly alter parasite pathogenicity for BALB/c mice. Although these ROPs did not seem to be essential for the acute infectious stage of type I *T. gondii* in the mouse model, they might have different functions in other intermediate hosts or play different roles in other life cycle forms of this parasite due to the different expression patterns; this warrants further investigations.

## Introduction

*Toxoplasma gondii*, an obligate intracellular protozoan pathogen, has the ability to infect a wide range of warm-blooded animals and humans (Pappas et al., 2009; Dubey, 2010; Andiappan et al., 2014; Chemoh et al., 2015; Liu et al., 2015). This parasite has a great medical importance because infection of immunocompromised individuals, including AIDS, cancer, and transplant patients, can lead to serious illness. Also, primary infection during pregnancy can lead to spontaneous abortion, fetal death, and significant congenital complications, such as brain and visual impairment (Pappas et al., 2009; Dubey, 2010; Andiappan et al., 2014; Chemoh et al., 2015; Liu et al., 2015). One of the most remarkable features of *T. gondii* is its ability to infect another eukaryotic cell and hijack the functions of such a host cell once within. Intracellular survival of this parasite is critically dependent on its ability to actively invade surrogate host cell, establish a replication-permissive vacuole and avoid host cell immune defenses (Melo et al., 2011; Hunter and Sibley, 2012).

Another important feature of *T. gondii* is the dramatic difference between strains in terms of their virulence in animal models (Su et al., 2003, 2012; Lorenzi et al., 2016) and also in human infections (Niedelman et al., 2012). This explains why disease that *T. gondii* causes in humans ranges from essentially asymptomatic to debilitating or even life threatening. There are likely many reasons for these differences, but evidence from animal studies and clinical studies in humans indicate that strain differences in the parasite likely play a major role and a number of genes were found to vary between strains and interact with the innate immunity in different ways (Saeij et al., 2006; Taylor et al., 2006; Behnke et al., 2011; Reese et al., 2011; Niedelman et al., 2012). It is intriguing that the major virulence determinants of this parasite are the effector secretory proteins derived from the apical highly specialized secretory organelles known as rhoptries (ROPs). Rhoptries contain many parts of the invasion machinery, located within the rhoptry necks and known as RONs, and a collection of effector proteins known as ROPs that are located within the rhoptry bulbs and intersect several host signaling pathways key to the pathogenesis and immune evasion, such as STAT signaling and immunity-related GTPases (IRGs or p47 GTPases) (Bradley and Sibley, 2007; Boothroyd and Dubremetz, 2008; Melo et al., 2011; Hunter and Sibley, 2012). Many ROPs contain a conserved serine/threonine protein kinase domain and may function as kinases or pseudokinases, which include only part of the catalytic triad, possibly modifying host cell pathways by phosphorylation of specific targets (Melo et al., 2011; Hunter and Sibley, 2012).

Comparative genomic and phylogenetic analyses revealed approximately 50 rhoptry kinases and pseudokinases (ROPKs) in *T. gondii* genome (Bradley et al., 2005; Peixoto et al., 2010; Talevich and Kannan, 2013). These ROPKs are polymorphic and can account for much of the differences observed in the virulence of different *T. gondii* strains. Some ROPKs have been shown to play roles in *T. gondii* virulence, establishing chronic *T. gondii* infection and manipulating the host innate immune response (Saeij et al., 2006; Taylor et al., 2006; Behnke et al., 2011; Butcher et al., 2011; Reese et al., 2011; Hunter and Sibley, 2012; Camejo et al., 2014; Etheridge et al., 2014; Fox et al., 2016; Jones et al., 2016; Kim et al., 2016). For instance, ROP5, ROP17, and ROP18 forming ROP5/17/18 complex function as virulence factors to prevent immune related GTPases accumulation at the parasitophorous vacuole (PV) (Saeij et al., 2006; Taylor et al., 2006; Behnke et al., 2011; Reese et al., 2011; Etheridge et al., 2014). ROP54, a type II *T. gondii* virulence factor, modulates the innate immune loading of GTP-binding protein 2, which does not act by interacting with the ROP5/17/18 complex (Kim et al., 2016). ROP16 can inhibit the production of the IL-12 in a parasite strain type dependent manner via activation of the host transcription factors STAT3 and STAT6 (Butcher et al., 2011) and can mediate human neuroblastoma SH-SY5Y apoptosis and cell cycle arrest in G1 phase by affecting the expression of Bax/Bcl-2 and p21/CDKs (Chang et al., 2015). More recently, deletion of 26 ROP gene loci encoding for 31 unique ROPK proteins in the less virulent *T. gondii* type II strain showed that most ROP proteins are essential for the establishment of chronic infection and that while some ROPs can serve as virulence factors by resisting innate immunity and other ROPs can influence chronic infection without affecting virulence (Fox et al., 2016), indicating the diverse roles that ROPs can play in mediating the parasite interaction with host.

Given the polymorphism of most ROPs and distinct biological and pathogenic traits of *T. gondii* strains, it is likely that these rhoptry paralogs have different functions in different strains of *T. gondii* (Su et al., 2012; Behnke et al., 2015; Lorenzi et al., 2016). Hence, determining how differences between *T. gondii* strains result in different pathogenicity in their hosts may help optimize treatment for individual patients. The contributions of most rhoptry kinases to host–pathogen interaction processes have been investigated in the less virulent type II strain, but the functions of most rhoptry kinases in the highly virulent type I strains remain largely unknown. Therefore, in this work, we utilized CRISPR-Cas9 technology to disrupt 15 ROP genes, namely *rop10, rop11, rop15, rop20, rop23, rop31, rop32, rop33, rop34, rop35, rop36, rop40, rop41, rop46*, and *rop47* in the high virulent *T. gondii* RH strain (type I). Then, we evaluated the capacity of mutant strains of *T. gondii* deficient in these individual ROPs to induce lesions *in vitro* growth and to cause illness in experimentally infected BALB/c mice.

## Materials and Methods

### Parasites and Cell Culture

Tachyzoites of *T. gondii* (strain RH; type I) were grown *in vitro* in human foreskin fibroblast (HFF, ATCC^®^ SCRC-1041^TM^) in a humidified incubator with 5% CO_2_ at 37°C. HFF cell culture was maintained by biweekly passage in Dulbecco’s modified Eagle medium (DMEM) supplemented with 10% fetal bovine serum (FBS) and 10 μg/mL gentamicin. Infected cells were syringe-lysed using a 27-gauge needle to release tachyzoites from host feeder cells into DMEM medium and then filtered using a 5 μm pore size Millipore filter.

### Construction of ROP Knockouts by CRISPR-Cas9

The development of bacterial CRISPR-Cas9 system for use as a genome-editing tool has accelerated the pace of reverse genetic studies including rapid gene knockout (KO) and targeted modifications in *T. gondii* (Shen et al., 2014; Wang et al., 2016a). Hence, in this study targeted genetic manipulation of *T. gondii* was performed using CRISPR-Cas9 technology to disrupt 15 previously defined ROP genes, namely *rop10, rop11, rop15, rop20, rop23, rop31, rop32, rop33, rop34, rop35, rop36, rop40, rop41, rop46*, and *rop47*. The latter gene was annotated as a putative polo kinase in *T. gondii* genome and was proposed as *rop47* (Talevich and Kannan, 2013). Details of all plasmids, primers and sgRNAs used in this study are provided in the Supplemental Material (Table S1). The CRISPR-Cas9 expression plasmids were constructed using methods previously described (Shen et al., 2014; Wang et al., 2016b). Briefly, sgRNAs were engineered into pSAG1::CAS9-U6::sgUPRT (Addgene #54467) using the Q5 Mutagenesis Kit (NEB). Resistance cassettes DHFR^∗^-Ts were amplified from the plasmid pUPRT-DHFR-D using Q5 DNA Polymerase (NEB). Freshly harvested tachyzoites were obtained, purified as described above and 40-μg CRISPR-Cas9 plasmids were cotransfected with the 10-μg purified DHFR^∗^-Ts amplicons. Successfully transgenic parasites were obtained by selection with 3 μM pyrimethamine (Sigma Aldrich, St. Louis, MO, USA) and single clones were screened using methods previously described (Shen et al., 2014; Wang et al., 2016b). Diagnostic PCRs were carried out with genome DNA as template to confirm the insertion of DHFR^∗^-Ts at the sgRNA-targeted coding region in the 15 endogenous ROP loci.

### RT-PCR

First-strand cDNA was generated from total RNA of *T. gondii* using a PrimeScript^TM^ 1st Strand cDNA Synthesis Kit (TaKaRa). cDNAs from specific ROP KOs and wild type (WT) RH strain were tested by RT-PCR to amplify a small fragment around the insertion site in each targeted gene using specific primers listed in the Supplemental Material (Table S1).

### Plaque Assay

For parasite plaque assays, 200 *T. gondii* tachyzoites of ROP-deficient or WT strains were inoculated into confluent HFF monolayers grown in six-well cell culture plates. The infected cell cultures were monitored microscopically over 7 days after infection. Then, the cells were fixed with 4% paraformaldehyde and stained for 10 min with 2% crystal violet to visualize and count plaques formed by the growing parasites by using inverted microscope (Wang et al., 2016b; Zhang et al., 2016).

### Quantification of Ionophore-Induced Parasite Egress

Briefly, 1 × 10^5^ freshly harvested tachyzoites were seeded into confluent HFF monolayers in 12-well cell culture plates and were incubated for 30–36 h. Then, cell monolayers were washed with sterile phosphate-buffered saline (PBS) followed by incubation with 3 μM calcium ionophore A23187 (Sigma) diluted in DMEM or an equivalent amount of DMSO as a solvent control for 10 min. Live videos were performed to document the progress of parasite egress as previously described (McCoy et al., 2012; Wang et al., 2016b).

### Infectivity Studies with Mice

Mice (female inbred BALB/c, 7 weeks old) were purchased from Center of Laboratory Animals, Lanzhou Institute of Biological Products, Lanzhou, China. Mice were housed (five mice/cage) under a 12-h light:dark cycle (8:00–20:00) and under specific-pathogen-free conditions within the animal care facility until the end of the experiment. All mice were handled in strict accordance with the Good Animal Practice Requirements of the Animal Ethics Procedures and Guidelines of the People’s Republic of China. The study was approved by the Animal Ethics Committee of Lanzhou Veterinary Research Institute, Chinese Academy of Agricultural Science. Approximately, 200 freshly harvested tachyzoites were intraperitoneally injected into each BALB/c mice (10 mice/parasite strain) and the animals were monitored daily for signs of illness. Animal experiment began after 1 week of habituation and all efforts were made to minimize animal suffering.

### Bioinformatic Analyses of *T. gondii* Rhoptries Proteins (ROPs)

Genomic data (the number of exons, transmembrane domains, and signal peptide) and transcriptomic data (cell cycle expression profiles, oocyst, tachyzoite, and bradyzoite developmental expression profiles) of 15 ROPs were obtained from http://ToxoDB.org (Gajria et al., 2007). Single nucleotide polymorphisms (SNP) from the coding sequence of three *Toxoplasma* strains (GT1, ME49, and VEG) were analyzed by the Clustal W program in the MEGA 5.0 (Tamura et al., 2011).

## Results and Discussion

We have investigated the role of 15 ROP genes (*rop10, rop11, rop15, rop20, rop23, rop31, rop32, rop33, rop34, rop35, rop36, rop40, rop41, rop46*, and *rop47*) in the type I *T. gondii* (RH strain), to determine whether they play critical roles in *T. gondii*-host interaction, on replication in human cells and virulence in the BALB/c mice.

### Generation of ROP Knockouts in Type I RH Strain

**FIGURE 1:**
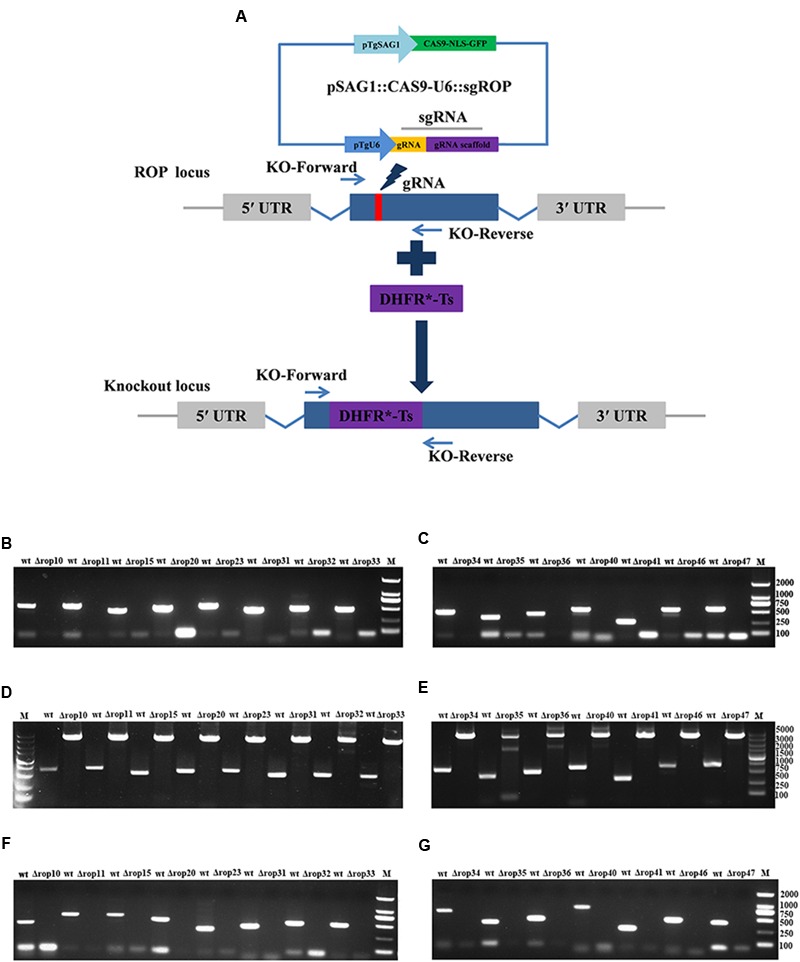
***Toxoplasma gondii* rhoptry organelle proteins (ROP) knockout (KO) mutant construction. (A)** Schematic illustration of CRISPR-Cas9-mediated gene KO by insertion of the pyrimethamine-resistant DHFR^∗^-Ts in the ROPs coding sequence. **(B–E)** KO forward and KO reverse primers were used to amplify the small fragment with 30 s extension time **(B,C)** or the large fragment with 4 min extension time **(D,E)**. Diagnostic PCR demonstrates DHFR^∗^-Ts integration and ROP genes disruption in a representative clone compared with wild-type RH strain. **(F,G)** RT-PCR screening of ROP gene KOs. Small fragment around the insertion site for each ROP gene was not detected by RT-PCR demonstrating that the coding sequences were disrupted.

To assess the contribution of 15 ROPs to *T. gondii* infection, CRISPR-Cas9 system was used to disrupt these genes via the insertion of DHFR^∗^-Ts at the sgRNA-targeted coding region in the 15 endogenous ROP loci (**Figure 1A**). Diagnostic PCR with expected small KO strains could be yielded in the WT RH strains, while a large fragment containing about 3.3 kb DHFR^∗^-Ts amplification was not amplified in the KO strains with 30 s extension time (**Figures 1B,C**), but could be yielded with 4 min extension time (**Figures 1D,E**). Also, mRNA from the targeted sites was not detected by RT-PCR in the KO strains for each of the ROP genes (**Figures 1F,G**). Insertion of a large DHFR^∗^-Ts fragment in the interested coding sequence caused shift mutations followed by gene disruption. The generation of the 15 ROPs KOs was successfully and rapidly achieved.

### Phenotypic Analyses of ROPs Knockouts *In vitro*

To test the phenotypes of ROP KOs during the parasite’s lytic cycle, plaque assay, an assay that captures invasion, egress, motility and replication was performed to compare the intracellular growth of mutants to the WT RH strain. Over a 7-day period of the parasite lytic cycle, the number and the size of individual plaques developed by each strain were recorded. There was no significant difference between all ROP KOs and the RH WT strain (**Supplementary Figure S1**). We also evaluated whether there was any alteration in ionophore-induced egress (IIE) induced by ROP gene deletion. Calcium ionophore was used to stimulate intracellular tachyzoites to rapidly egress from their PV at any stage during the lytic cycle given that the natural egress normally occurs when there are 64 or more parasites inside the PV (Black and Boothroyd, 2000; Black et al., 2000). Based on time-lapse imaging analysis of infected cultures stimulated with 3 μM calcium ionophore A23187 or equivalent amount of DMSO as control over 10 min revealed that all 15 ROP KO strains and the WT RH strain rapidly egressed from their PVs within 4 min after the stimulation with 3 μM calcium ionophore A23187 (**Supplementary Figure S2**), while all 15 ROP KO strains and the WT RH strain were still in their PVs after the stimulation with equivalent amount of DMSO. These findings show that infection of HFF cells with these ROP KO strains did not entail a significant reduction in parasite multiplication or any significant alteration in the parasite’s growth pattern. This result was not surprising, as some ROP genes have previously been shown to be not required for the parasite’s ability to invade and colonize HFF cells, such as ROP5 and ROP18 (Saeij et al., 2006; Taylor et al., 2006; Behnke et al., 2011; Reese et al., 2011; Etheridge et al., 2014; Fox et al., 2016).

### Characterization of ROP Knockouts *In vivo*

**FIGURE 2:**
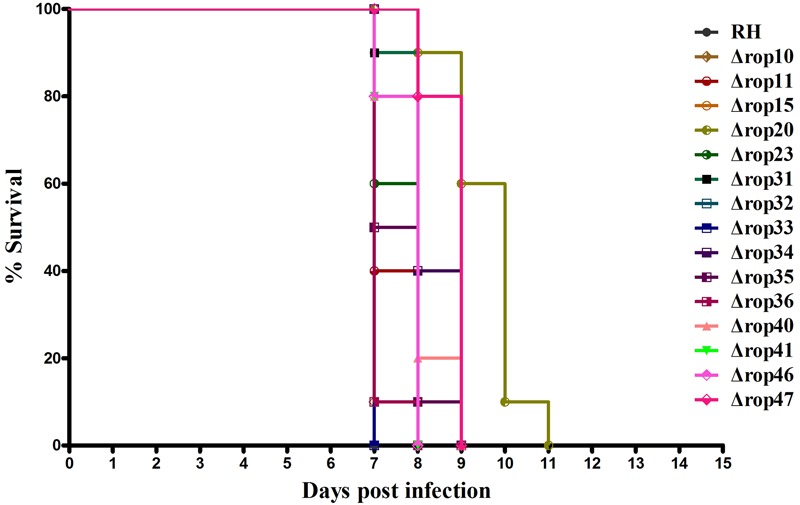
**Survival curves of BALB/c mice infected with *T. gondii* (RH) wild-type strain or ROP KO strains.** The various groups of BALB/c mice were challenged with 200 tachyzoites of the indicated strains. Each group was composed of 10 mice and survival time was monitored daily for 11 days after challenge.

Even though ROP5, ROP17, and ROP18, as well as other ROPs have been shown as important virulence factors, deletion of these genes did not or slightly affect the parasite infection cycle *in vitro*. We were also interested in whether deletion of these ROPs in RH strain would affect its virulence as described previously in ROP5-, ROP17- and ROP18-deficient strains (Saeij et al., 2006; Taylor et al., 2006; Behnke et al., 2011; Reese et al., 2011; Etheridge et al., 2014). Approximately, 200 WT RH tachyzoites or an equal number of the generated ROP KOs were used to infect BALB/c mice. Overall, deletion of the 15 ROPs in type I *T. gondii* RH strain did not cause significant impact on the parasite virulence in mice. Compared to mice infected with WT strain all mice infected with ROP KO strains showed similar mortality or slightly reduced virulence with mice dying between days 7 and 11 post infection (**Figure 2**). There may be some level of function redundancy in these ROPs, as some ROPs in type II strain both have the ability to increase cyst burdens in order to establish chronic infection in mice (Fox et al., 2016; Jones et al., 2016). Perhaps, the effects of deleting these ROP genes in the *T. gondii* RH did not result in phenotype changes due to function redundancy, or alternatively these genes may play no significant role in the virulence in mouse model that we have tested. For example, these ROPs genes may serve as virulence factors in other intermediate hosts. However, this question is quite challenging because *T. gondii* can infect almost all warm-blooded animals.

### Sequence and Expression Analyses of ROPs in *T. gondii*

**FIGURE 3:**
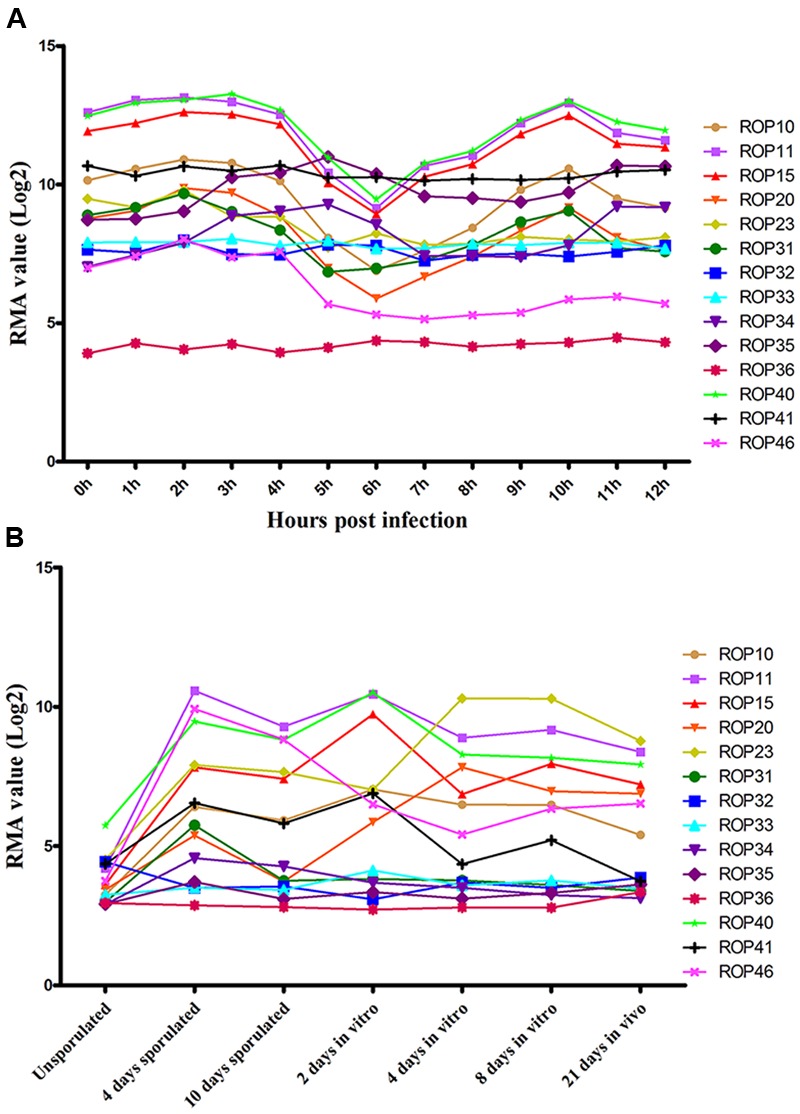
**Expression analysis of ROPs in *T. gondii* (Note: data were not available for ROP47). (A)** *Toxoplasma* RH cell cycle microarray expression data for ROP genes were obtained from http://ToxoDB.org. h, the time post-thymidine release; S, synthesis; M, mitosis; C, cytokinesis; G1, G1 phase of cell cycle; RMA, robust multiarray average algorithm. **(B)** Transcriptomic expression data of *Toxoplasma* ROP genes of the parasite developmental stages, oocyst, tachyzoite, and bradyzoite, of type II strain M4 were obtained from http://ToxoDB.org. Sources of the RNAs: oocysts recovered from cat feces included day 0 (unsporulated) oocysts, day 4 post-induction of sporulation (4 days sporulated) and day 10 post-induction of sporulation (10 days sporulated). *In vitro*, 2 dpi tachyzoite, 4 and 8 dpi bradyzoite samples (2 days *in vitro*, 4 days *in vitro*, 8 days *in vitro*) are from separately infected cultures of HFF cell. *In vivo*, 21 dpi bradyzoite cysts (21 days in *vivo*) harvested from the brains of mice infected with oocysts.

Previous studies have identified ∼50 ROPs in *T. gondii* genome and some of these, such as ROP5, ROP16, ROP17, and ROP18, are typically expressed in a characteristic cell cycle-dependent profile where their mRNA expression levels were found to increase to maximum 2–3 h post-thymidine release (S to M phase) and then dramatically decrease in early G1 (minimum 6 h) followed by a rise again in the next S phase (peak 10 h) (Behnke et al., 2010; Camejo et al., 2014). However, some ROPs did not exhibit any obvious cyclical expression profile, such as ROP21, ROP27, and ROP28 (Jones et al., 2016). To examine the expression of the 15 ROP genes tested in the present study during cell cycle expression, we analyzed microarray data obtained from ToxoDB^1^ (Behnke et al., 2010). Profiling the cell cycle expression profiles of the 15 ROPs revealed that several ROPs (ROP10, ROP11, ROP15, ROP20, and ROP40) are expressed in a characteristic cell cycle profile while most are not (ROP23, ROP31, ROP32, ROP33, ROP34, ROP35, ROP36, ROP41, and ROP46) (**Figure 3A**). Other typical bioinformatic features for ROPs are expressed from single exon genes and have a predicted signal peptide that mediates their trafficking to the parasitophorous vacuole membrane (PVM) or the host cell (Camejo et al., 2014; Lorenzi et al., 2016). However, we found that some of these ROPs are encoded by multi-exons and some do not have a predicted signal peptide (**Table 1**). Our analysis also suggests that some annotated ROPs are not consistent with and may have different functions from the canonical ROPs. We also analyzed the transcriptomic levels of 15 ROP genes during various life cycle forms of the parasite^2^ based on a previous published transcriptomic data (Fritz et al., 2012). This analysis revealed the transcriptomic expression level of some ROPs (ROP32, ROP33, ROP34, ROP35, and ROP36) to be constitutively expressed while others (ROP10, ROP11, ROP15, ROP20, ROP23, ROP31, ROP40, ROP41, and ROP46) are differentially expressed during *Toxoplasma* development (**Figure 3B**). The presence of some ROPs that are differentially expressed during the parasite developmental cycle and other ROPs that are expressed throughout the parasite life cycle indicate that these ROPs may play different roles during different life cycle stages, such as the sexual phase in the cat intestine or during oocyst development. However, the RH strain lost its capacity to complete the life cycle in cat (Frenkel et al., 1976). Using CRISPR-Cas9 in other cat-competent type I *T. gondii* strains, such as the GT1, would allow for testing the functions of ROPs during sexual development or during oocyst development.

**Table 1:** Characterization sequence features of rhoptry organelle proteins (ROPs) of *Toxoplasma gondii.*

Name	Gene ID	Product description	Exons	Transmembrane domains	Active- or pseudo-kinases	SNPs^∗^	Predicted signal peptide
ROP10	TGGT1_315490	Rhoptry protein ROP10 (ROP10)	1	0	Active-kinase	4	Yes
ROP11	TGGT1_227810	Rhoptry kinase family protein ROP11 (incomplete catalytic triad)	1	0	pseudo-kinases	12	Yes
ROP15	TGGT1_211290	Rhoptry protein ROP15 (ROP15)	6	0	Active-kinase	9	Yes
ROP20	TGGT1_258230	Rhoptry kinase family protein ROP20	1	1	Active-kinase	23	Yes
ROP23	TGGT1_239600	Rhoptry kinase family protein ROP23 (incomplete catalytic triad)	1	0	pseudo-kinases	3	Yes
ROP31	TGGT1_258800	Rhoptry kinase family protein ROP31	1	1	Active-kinase	1	Yes
ROP32	TGGT1_270920	Rhoptry kinase family protein ROP32	2	0	Active-kinase	1	Yes
ROP33	TGGT1_201130	Rhoptry kinase family protein ROP33	3	1	Active-kinase	13	Yes
ROP34	TGGT1_240090	Putative rhoptry kinase family protein ROP34	2	0	Active-kinase	16	No
ROP35	TGGT1_304740	Rhoptry kinase family protein ROP35	3	0	Active-kinase	14	No
ROP36	TGGT1_207610	Rhoptry kinase family protein ROP36 (incomplete catalytic triad)	1	0	pseudo-kinases	12	Yes
ROP40	TGGT1_291960	Rhoptry kinase family protein ROP40 (incomplete catalytic triad)	3	0	pseudo-kinases	12	Yes
ROP41	TGGT1_266100	Rhoptry kinase family protein ROP41	1	0	Active-kinase	6	No
ROP46	TGGT1_230470	Putative rhoptry kinase family protein ROP46	2	0	Active-kinase	2	No
ROP47	TGGT1_252500	Polo kinase	1	0	pseudo-kinases	18	Yes

*^∗^SNPs: single nucleotide polymorphisms from the coding sequence of three *Toxoplasma* strains (GT1, ME49, and VEG).*

## Conclusion

*Toxoplasma* synthesizes and exports several proteins, such as rhoptry kinases (ROPs), to the host cells to ensure parasite’s invasion and survival within the host. The present study investigated the contribution of 15 ROPs to *T. gondii* infection process *in vitro* and *in vivo* by disrupting individual ROP genes using the CRISPR-Cas9 approach. Our results showed that deletion of these ROPs does not seem to interfere with the parasite ability growth *in vitro* or to induce illness in experimentally infected BALB/c mice. Infection of BALB/c mice with the KO mutants demonstrated that the deletion of single ROP genes was insufficient to induce a high level of attenuation compared with the parental parasite strain. The different expression patterns of some ROPs suggest that they may have different functions during various stages of the parasite’s life cycle. Elucidation of the roles of these ROPs in the various life cycle stages of *T. gondii* and in other strains is needed.

## Ethics Statement

The study was approved by the Animal Ethics Committee of Lanzhou Veterinary Research Institute, Chinese Academy of Agricultural Science.

## Author Contributions

X-QZ and S-YH designed this study and critically revised the manuscript. J-LW, T-TL, and KC performed the experiments, analyzed data and drafted the manuscript. HE, W-NZ, and D-MY participated in manuscript revision. All the authors read and approved the final manuscript.

## Conflict of Interest Statement

The authors declare that the research was conducted in the absence of any commercial or financial relationships that could be construed as a potential conflict of interest.

## Footnotes

1 http://ToxoDB.org

2 http://ToxoDB.org

## Supplementary Material

FIGURE S1 **Phenotype analyses of wild type RH and ROP-deficient parasites.** Two hundred freshly harvested *Toxoplasma* tachyzoites of wild type RH and ROP-deficient parasites were seeded in six-well plates. After 7 days of growth, the plaque numbers were recorded. All parasite strains were performed three times independently, each with triplicates.

FIGURE S2 **Egress phenotype of the parental strain RH and one of ROP mutants (ROP10).** Live time-lapse microscopy of parental parasite **(A)** and one of ROP mutants (ROP10) **(B)** show both parental parasite and ROP10 mutant can activate egress within 2 min after addition of 3 μM calcium ionophore A23187. The egress phenotype of other ROP mutants is not shown due to the similar results.
