# Supplementary figures and images for: Functional Characterization of Rhoptry Kinome in the Virulent Toxoplasma gondii RH Strain
Source: Front Microbiol. 2017 Jan 24;8:84. doi: 10.3389/fmicb.2017.00084 (PMC5258691; doi:10.3389/fmicb.2017.00084)

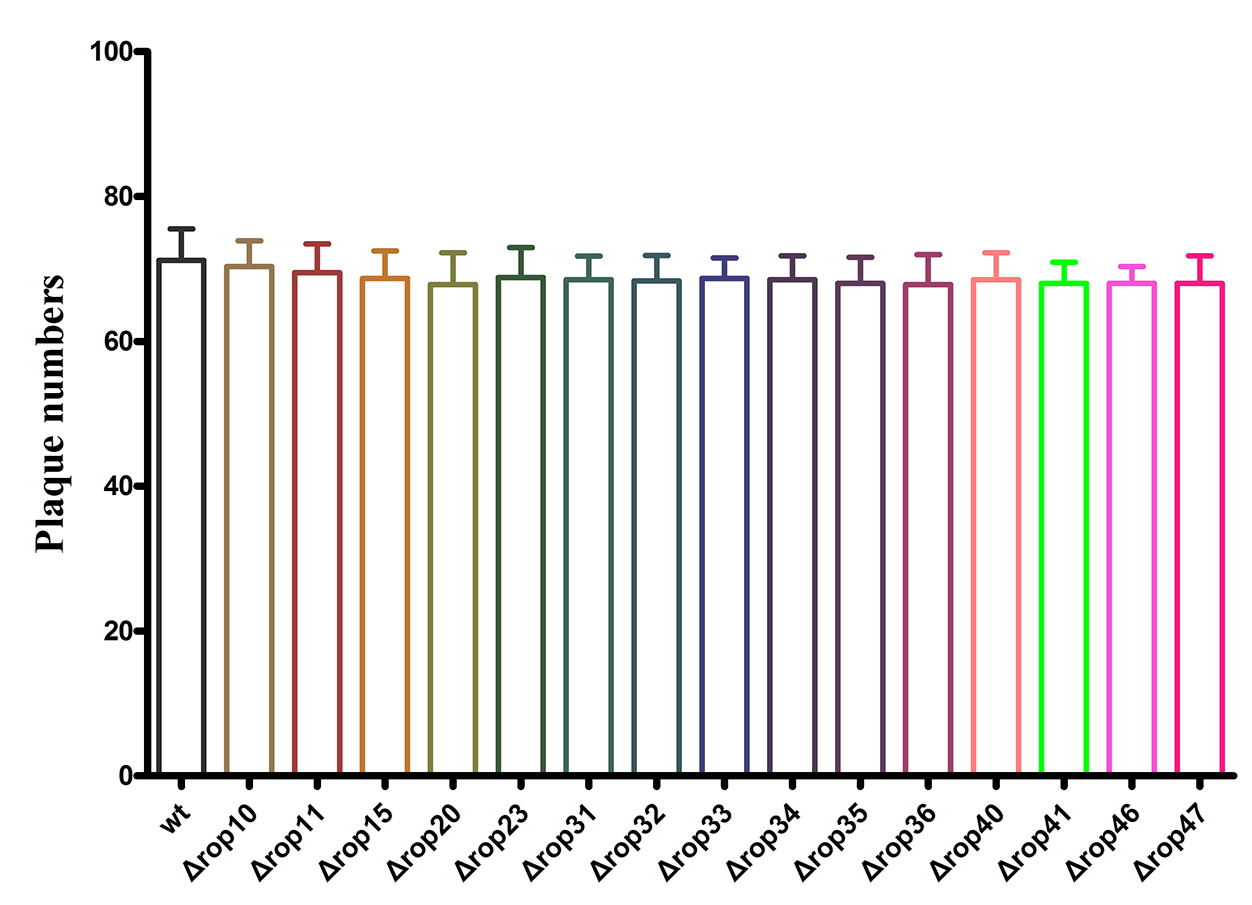

Supplement: FIGURE S1 — Phenotype analyses of wild type RH and ROP-deficient parasites. Two hundred freshly harvested Toxoplasma tachyzoites of wild type RH and ROP-deficient parasites were seeded in six-well plates. After 7 days of growth, the plaque numbers were recorded. All parasite strains were performed three times independently, each with triplicates. [file Image_1.TIF]

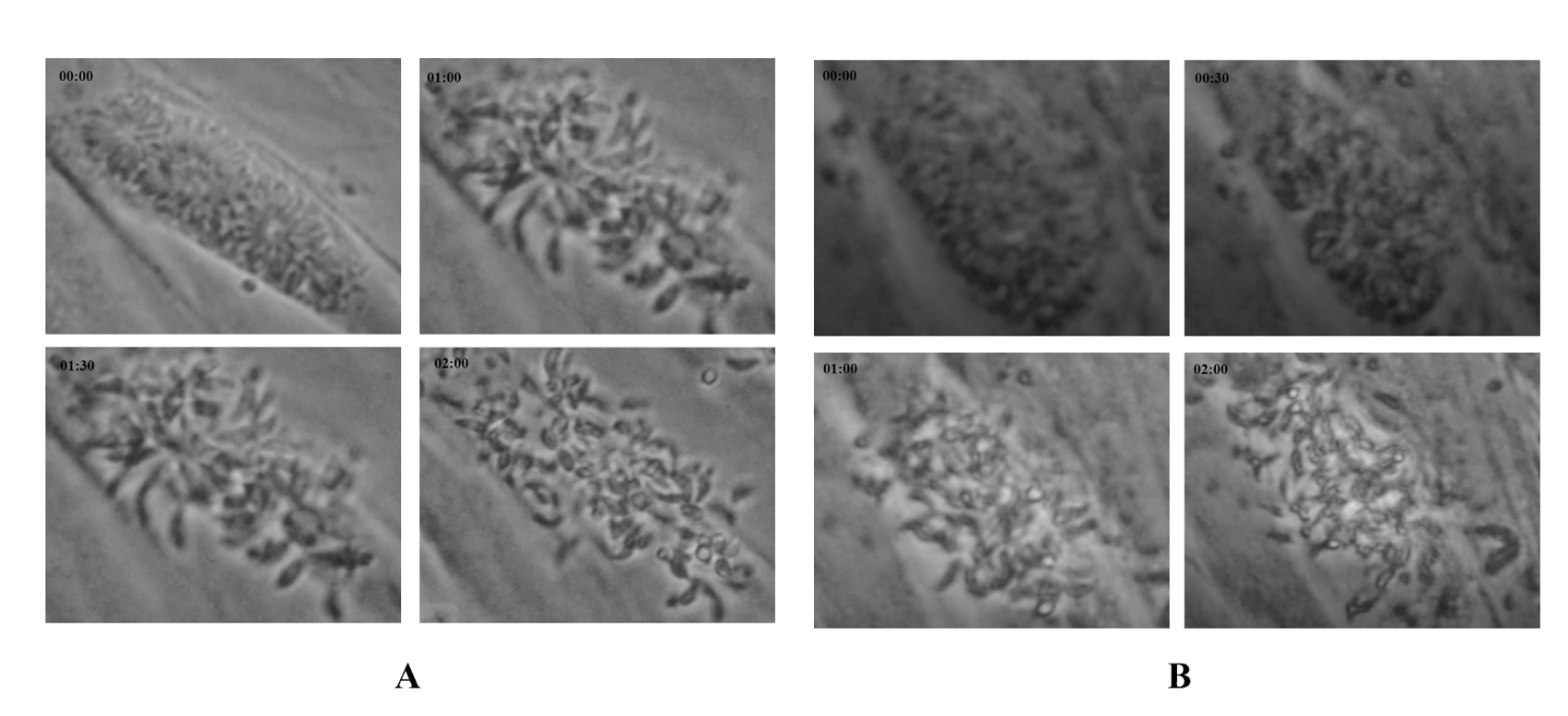

Supplement: FIGURE S2 — Egress phenotype of the parental strain RH and one of ROP mutants (ROP10). Live time-lapse microscopy of parental parasite (A) and one of ROP mutants (ROP10) (B) show both parental parasite and ROP10 mutant can activate egress within 2 min after addition of 3 μM calcium ionophore A23187. The egress phenotype of other ROP mutants is not shown due to the similar results. [file Image_2.TIF]
