# Supplementary material for: Functional Characterization of Rhoptry Kinome in the Virulent Toxoplasma gondii RH Strain
Source: Front Microbiol. 2017 Jan 24;8:84. doi: 10.3389/fmicb.2017.00084 (PMC5258691; doi:10.3389/fmicb.2017.00084)
Supplement: Supplementary file 3 [file Table_1.DOC]

**TABLE S1｜The information of ROP genes, sgRNAs and identification primers used in this study**

| **Name** | **sgRNA** | **KO-Forward primer** | **KO-Reverse primer** | **RT-Forward primer** | **RT-Reverse primer** |
| --- | --- | --- | --- | --- | --- |
| ROP10 | GACATCTTCGCTACCGCCCG | TCGTCTCTTTTTCGACTCGTT | TCCGTCAGATTCTTCTTCCCT | ATGGGACGACCCAGGTGGCCTC | TCCGTCAGATTCTTCTTCCCT |
| ROP11 | GGGGCTTTGCGCTACCCTTC | CTGGTTTTCGAGGTTGAGGAA | TGAAGGTGACAGACGGGAGTT | CTGGTTTTCGAGGTTGAGGAA | TGAAGGTGACAGACGGGAGTT |
| ROP15 | GAAGGGGCGGCGAAGAGCTC | GATTCTGCTGTTCTATCGTTC | TGTTTGTCTTTTGCCGTCTTA | TAACGTGGATGTTCCCTAGATG | GTCAGAGGCTTCGTGATTGC |
| ROP20 | GGAGAAGAAATTGGAGACGG | CAGCTCAGTCCTTGCCCCCAC | ACCGCAGCCACAACGACATTC | CAGCTCAGTCCTTGCCCCCAC | ACCGCAGCCACAACGACATTC |
| ROP23 | GACATCTCCACGTTCAGACG | ACATAAGGAAGGAAGCCAGCA | TCGAACAATGACTCCGAAAGG | ATGGAAAAGATCCTGTGGGCGGCG | TCGAACAATGACTCCGAAAGG |
| ROP31 | GAAGAATCTGCACTCGAGAC | GCTGCTGAGCGTCCCTGTAAG | GCCAAGCCAAGCGGGCGAAAT | ATGCGAGCCCAGGTGGCACTGTG | GCCAAGCCAAGCGGGCGAAAT |
| ROP32 | GGACTGTGAATAGACCCGAC | CTGTGCTGCTATTCCGTGGGG | CGTTGACGCTGTCGGTTTGGG | CTGTGCTGCTATTCCGTGGGG | CGTTGACGCTGTCGGTTTGGG |
| ROP33 | GAAGCGGAGACAGAAAATGC | CTTCTCCACGCCGCTTCCCAT | TCGCCTGACGCAGTGTCGCTC | CTTCTCCACGCCGCTTCCCAT | TCGCCTGACGCAGTGTCGCTC |
| ROP34 | GCTCGCGAATCGCGAACCAC | CAACCAACTTTCTTTTTTCTT | GACTGAGTCTTGATTTGCACT | GTCCTTGCGTGACTTCCACATTC | ACCAGGTACAGGACTCGCTTCG |
| ROP35 | GCGATGTGTCTGGCATCGGA | TGGGATCTTTTGTTTCTTTCC | CCATTGTGTGCTTCTCCTCTA | TGGGATCTTTTGTTTCTTTCC | CCATTGTGTGCTTCTCCTCTA |
| ROP36 | GGCAGAAGCGAAACGCCCTG | GGTCGCTGTTCATTTTTGTGT | CTTCTTCTATCTCGTTGCCTT | GGTCGCTGTTCATTTTTGTGT | CTTCTTCTATCTCGTTGCCTT |
| ROP40 | GTCCCCCCTCTGGTATTCCA | AATAGAATGCCTGGTGCTTGT | GCTGGTGTCTGCTGTTTGACG | TGACGGGACTACCGCAACC | TTACCATCCGCAACACCAAAT |
| ROP41 | GAGGAAAGCGTTTCGCTGGA | GGACCTGTGTCGAACACCTCA | CAGCAGCAATCGCACCTTTAT | GGACCTGTGTCGAACACCTCA | CAGCAGCAATCGCACCTTTAT |
| ROP46 | GCACTGAGAAGGATGCTGAA | TAAAGAACTCGCACCAAAAAG | AGAGACAGGGGTCTAACAGCA | GCACGACGGGCAACGTATA | GAGCAGCAGGATCGGAAGC |
| ROP47 | GACGTGCGGCCATCGCGGTC | CAACTGGCAAATCCGTTCCTT | CAGAGTCCTGTGTGTCACCCT | ATGAGGCGTTCGAGGTCAAAATACC | CAGAGTCCTGTGTGTCACCCT |
